# Exploring the chaotic structure and soliton solutions for (3 + 1)-dimensional generalized Kadomtsev–Petviashvili model

**DOI:** 10.1038/s41598-024-66765-9

**Published:** 2024-07-09

**Authors:** Muhammad Nadeem, Ding Jingxia, Kalim U. Tariq, Yahya Alsayaad

**Affiliations:** 1 School of Mathematics and Statistics, Qujing Normal University, Qujing, 655011 China; 2 Department of Mathematics, Mirpur University of Science and Technology (MUST), Mirpur, AJK 10250 Pakistan; 3 Department of Physics, Hodeidah University, Al-Hudaydah, Yemen

**Keywords:** Generalized KP model, Modified Sardar subequation technique, Invariant solutions, Soliton solutions, Chaotic structure, Applied mathematics, Computational science, Software

## Abstract

The study of the Kadomtsev–Petviashvili (KP) model is widely used for simulating several scientific phenomena, including the evolution of water wave surfaces, the processes of soliton diffusion, and the electromagnetic field of transmission. In current study, we explore some multiple soliton solutions of the (3+1)-dimensional generalized KP model via applying modified Sardar sub-equation approach (MSSEA). By extracting the novel soliton solutions, we can effectively obtain singular, dark, combo, periodic and plane wave solutions through a multiple physical regions. We also investigate the chaotic structure of governing model using the chaos theory. The behavior of the collected solutions is visually depicted to demonstrate the physical properties of the proposed model. The solutions obtained in this paper can expand the existing solutions of the (3+1)-dimensional KP model and enhance our understanding of the nonlinear dynamic behaviors. This approach allows for consistent and effective treatment of the computation process for nonlinear KP model.

## Introduction

Nonlinear evolution equations (NLEEs) are widely recognized for their important role in explaining nonlinear scientific events. Numerous fields are covered by the study of these equations, including fluid mechanics, solitary waves theory, optical fibers, water waves, chaos theory, hydrodynamics and turbulence theory, chemical engineering, and optical science^1–6^. In recent decades, there has been a surge in the amount of study done on these nonlinear equations to gain an understanding of their qualitative and quantitative properties^7,8^. An essential component of nonlinearity, the soliton pulse shows a perfect harmony between dispersion effects and nonlinearity. Recently, there has been an explosion in research on integrable properties for NLEEs^9,10^. Kumar et al.^11^ employed the generalized exponential rational function to derive the novel soliton solution of (2+1)-dimensional Konopelchenko-Dubrovsky system. Ma and Lee^12^ used the generalized rational function technique to obtain the solution of 3+1 dimensional Jimbo-Miwa problem. In^13^, the author analyzed the Hirota N-soliton conditions of three (2+1)-dimensional integrable equations. Wang and Li^14^ employed a bilinear technique to find novel exact solutions for the generalized (3+ 1)-dimensional Kadomtsev-Petviashvili problem. The NLEEs and their integrability have been studied using a wide range of effective techniques including, Inverse scattering technique^15^, Painlev analysis^16^, Generalized symmetry technique^17^, the extended sinh-Gordon equation expansion method^18^, Bäcklund transformation technique^19^, Pfaffian technique^20^, Hirota bilinear technique^21^, and many others^22–25^.

The KP model describes the evolution of nonlinear long waves with modest amplitude and gradual dependency on the transverse coordinate as^26^

1 $$\begin{aligned} ({\mathcal {P}}_{t}+6 {\mathcal {P}} {\mathcal {P}}_{x}+{\mathcal {P}}_{xxx})_{x}+a {\mathcal {P}}_{yy}=0, \end{aligned}$$

which shows a nonlinear partial differential equation in two spatial coordinates of *x*, *y* and one temporal coordinate of *t*. This equation allows to construct the fully integrable KP model when surface tension and viscosity effects are minimal. The modified KP model is as follows^27^

2 $$\begin{aligned} 4{\mathcal {P}}_{t}+ {\mathcal {P}}_{xxx} -6{\mathcal {P}}^2{\mathcal {P}}_{x}+6{\mathcal {P}}_{x}\partial ^{-1}_x {\mathcal {P}}_{y}+3{\mathcal {P}}^{-1}_{x} {\mathcal {P}}_{yy}=0. \end{aligned}$$

It was developed from research on how ion-acoustic waves spread in a plasma containing non-isothermal electrons^28^. It can explain the evolution of different solitary waves in multi-temperature electron plasmas, where two temperature electrons with different Maxwellian distributions expressed as two Boltzmann relations coexist with a collision-less multi-component plasma that contains cold ions. Recently, Wazwaz and Tantawy^29^ studied the (3+1)-dimensional generalized KP model in the following form

3 $$\begin{aligned} \frac{\partial ^2{\mathcal {P}}}{\partial t\, \partial x}+\frac{\partial ^2{\mathcal {P}}}{\partial t\, \partial y}+\frac{\partial ^2{\mathcal {P}}}{\partial t\, \partial z}+\frac{\partial ^4{\mathcal {P}}}{\partial x\, \partial x\, \partial x\, \partial y}-\frac{\partial ^2{\mathcal {P}}}{\partial z\, \partial z}+3 \frac{\partial }{\partial x}\left( \frac{\partial {\mathcal {P}}}{\partial x}\cdot \frac{\partial {\mathcal {P}}}{\partial y}\right) =0, \end{aligned}$$

whereas the soliton solutions are obtained in^30^.

This study aims to analyze the (3+1)-dimensional generalized KP model, which is an extension of the (2+1)-dimensional generalized KP model. We examine the modified Sardar sub-equation approach to obtain a variety of soliton solutions and phase portraits for the generalized KP model. Additionally, we employ the chaos theory to develop the phase analysis of a perturbed dynamical system. It is worth mentioning that generalized KP model (Eq. 3) has various applications in a wide range of physical phenomena^31^. The modified Sardar sub-equation method is a development of the sub-equation method that introduces specific modifications to enhance its efficacy in solving particular nonlinear partial differential equations (PDEs). These modifications involve customized transformations, the utilization of the Sardar sub-equation, and advanced integration techniques, resulting in improved accuracy and flexibility when compared to the general sub-equation method^32^. The specific soliton solutions for the generalized KP model have also been discovered inside a set of separate exact solutions^33^. However, we employ distinct approaches and phase analysis methods to acquire new soliton and dynamical characteristics. The MSSEA is limited to equations with very irregular or complicated nonlinearities that do not adhere to the usual forms, equations with non-polynomial or transcendental nonlinear components, equations containing plasma with several components and coupled reaction-diffusion theories. equations containing terms that are not local, and problems of nonlinear boundary conditions in domains that are not perfectly regular. The rest part of this article is designed as: “Methodology of the MSSEA”  presents a brief overview of the MSSEA for obtaining the precise solutions of generalized KP model. In “Mathematical analysis”, we implement the MSSEA and obtained the soliton solutions of the proposed model. In “Dynamical system”, we analyze the governing framework by transforming it into a dynamical structure and investigate the characteristics of the phase portrait. “Discussions and results” analyzes the physical reasoning for the obtained outcomes by utilizing visual representations of three-dimensional, two-dimensional, and density profiles. Lastly, “Conclusion” summarises our conclusion remarks.

## Methodology of the MSSEA

The MSSEA effectively used to solve NLEEs in various physical and mathematical problems. In nonlinear phenomena, the MSSEA improves on the conventional Sardar sub-equation technique by including more terms and clusters into the solution ansatz. The general structure of NLEEs is

4 $$\begin{aligned} {\mathcal {V}}({\mathcal {P}},~{\mathcal {P}}_x,~{\mathcal {P}}_t,~{\mathcal {P}}_{xx},{\mathcal {P}}_{x,z},~{\mathcal {P}}_{x,t},...)=0. \end{aligned}$$

Step 1. Utilize the transformation of complex wave form

5 $$\begin{aligned} {\mathcal {P}}={\mathcal {Q}}(\zeta ) \quad \quad \quad \zeta =-g_4 t+g_1 x+g_2 y+g_3 z. \end{aligned}$$

Using this transformation, Eq. (4) turns to ordinary differential equation such as,

6 $$\begin{aligned} {\mathcal {G}}({\mathcal {Q}},~{\mathcal {Q}}^{'},~{\mathcal {Q}}^{''},...)=0. \end{aligned}$$

in which ${\mathcal {G}}$ denotes the polynomial in ${\mathcal {Q}}(\zeta )$ and and prime shows the derivatives of $\zeta$.

Step 2. Consider the solution of Eq. (6) such as

7 $$\begin{aligned} {\mathcal {Q}}(\zeta )={\mathcal {H}}_{0}+\sum _{j=1}^{J}{\mathcal {H}}_{j}{\mathcal {J}}^{j}(\zeta ),~~~~~~~~~{\mathcal {H}}_{j} \ne ~0, \end{aligned}$$

where $${\mathcal {Q}}={\mathcal {Q}}(\zeta )$$ assures

8 $$\begin{aligned} {\mathcal {J}}'(\zeta )=\sqrt{l _2 {\mathcal {J}}(\zeta )^4+l _1 {\mathcal {J}}(\zeta )^2+l _0}, \end{aligned}$$

where the integers are $l _0\ne 1$, $l _1$ and $l _2\ne 0$. ${\mathcal {H}}_{0}$ and ${\mathcal {H}}_{1}$ are calculated, and ${\mathcal {H}}_{j}$ is invertible when it is zero. The balance principle rule is used to calculate the value of *J*. The Clusters to Eq. (8) are listed below.

Cluster 1. When $l _0=0,~l _1>0~ \text {and}~ l _2 ~\ne 0$, we acquire

9 $$\begin{aligned} {\mathcal {J}}_1(\zeta )= & {} \sqrt{-\frac{l _1}{l _2}} \text {sech}\Bigg (\sqrt{l _1} (\zeta +u )\Bigg ), \end{aligned}$$

10 $$\begin{aligned} {\mathcal {J}}_2(\zeta )= & {} \sqrt{-\frac{l _1}{l _2}} \text {csch}\Bigg (\sqrt{l _1} (\zeta +u )\Bigg ). \end{aligned}$$

Cluster 2. For constants $$k_{1}~\text {and}~k_{2}$$, When $$l _0=0,~l _1>0$$ and $$ l _2=+4 k_1 k_2$$, we acquire

11 $$\begin{aligned} {\mathcal {J}}_3(\zeta )=\frac{4 k_1 \sqrt{l _1}}{\Bigg (4 k_1^2-l _2\Bigg ) \sinh \Bigg (\sqrt{l _1} (\zeta +u )\Bigg )+\Bigg (4 k_1^2-l _2\Bigg ) \cosh \Bigg (\sqrt{l _1} (\zeta +u )\Bigg )}. \end{aligned}$$

Cluster 3. For constants $E_{1}~\text {and}~E_{2}$, When $l _0=\frac{l _1^2}{4 l _2},l _1<0 ~\text {and}~ l _2>0$, we acquire

12 $$\begin{aligned} {\mathcal {J}}_4(\zeta )= & {} \sqrt{-\frac{l _1}{2 l _2}} \tanh \Bigg (\sqrt{-\frac{l _1}{2}} (\zeta +u )\Bigg ), \end{aligned}$$

13 $$\begin{aligned} {\mathcal {J}}_5(\zeta )= & {} \sqrt{-\frac{l _1}{2 l _2}} \coth \Bigg (\sqrt{-\frac{l _1}{2}} (\zeta +u )\Bigg ), \end{aligned}$$

14 $$\begin{aligned} {\mathcal {J}}_6(\zeta )= & {} \sqrt{-\frac{l _1}{2 l _2}} \Bigg (\tanh \Bigg (\sqrt{-\frac{l _1}{2}} (\zeta +u )\Bigg )+i \text {sech}\Bigg (\sqrt{-2 l _1} (\zeta +u )\Bigg )\Bigg ), \end{aligned}$$

15 $$\begin{aligned} {\mathcal {J}}_7(\zeta )= & {} \sqrt{-\frac{l _1}{8 l _2}} \Bigg (\tanh \Bigg (\sqrt{-\frac{l _1}{8}} (\zeta +u )\Bigg )+\coth \Bigg (\sqrt{-\frac{l _1}{8}} (\zeta +u )\Bigg )\Bigg ), \end{aligned}$$

16 $$\begin{aligned} {\mathcal {J}}_8(\zeta )= & {} \frac{\sqrt{-\frac{l _1}{2 l _2}} \Bigg (\sqrt{e_1^2+e_2^2}-e_1 \cosh \Bigg (\sqrt{-2 l _1} (\zeta +u )\Bigg )\Bigg )}{e_1 \sinh \Bigg (\sqrt{-2 l _1} (\zeta +u )\Bigg )+e_2}, \end{aligned}$$

17 $$\begin{aligned} {\mathcal {J}}_9(\zeta )= & {} \frac{\sqrt{-\frac{l _1}{2 l _2}} \cosh \Bigg (\sqrt{-2 l _1} (\zeta +u )\Bigg )}{\sinh \Bigg (\sqrt{-2 l _1} (\zeta +u )\Bigg )+i}. \end{aligned}$$

Cluster 4. When $l _0=0,~l_1<0~\text {and}~l_2\ne 0$, we acquire

18 $$\begin{aligned} {\mathcal {J}}_{10}(\zeta )= & {} \sqrt{-\frac{l _1}{l _2}} \sec \Bigg (\sqrt{-l _1} (\zeta +u )\Bigg ), \end{aligned}$$

19 $$\begin{aligned} {\mathcal {J}}_{11}(\zeta )= & {} \sqrt{-\frac{l _1}{l _2}} \csc \Bigg (\sqrt{-l _1} (\zeta +u )\Bigg ). \end{aligned}$$

Cluster 5. When $$ l _0=\frac{l _1^2}{4 l _2},~l _1>0$$ and $$ l _2>0$$ and  $$E_1^2-E_2^2>0$$, we acquire

20 $$\begin{aligned} {\mathcal {J}}_{12}(\zeta )= & {} \sqrt{-\frac{l _1}{2 l _2}} \tan \Bigg (\sqrt{\frac{l _1}{2}} (\zeta +u )\Bigg ), \end{aligned}$$

21 $$\begin{aligned} {\mathcal {J}}_{13}(\zeta )= & {} -\sqrt{-\frac{l _1}{2 l _2}} \cot \Bigg (\sqrt{\frac{l _1}{2}} (\zeta +u )\Bigg ), \end{aligned}$$

22 $$\begin{aligned} {\mathcal {J}}_{14}(\zeta )= & {} -\sqrt{-\frac{l _1}{2l _2}} \Bigg (\tan \Bigg (\sqrt{2 l _1} (\zeta +u )\Bigg )-\sec \Bigg (\sqrt{2 l _1} (\zeta +u )\Bigg )\Bigg ), \end{aligned}$$

23 $$\begin{aligned} {\mathcal {J}}_{15}(\zeta )= & {} \sqrt{-\frac{l _1}{8 l _2}} \Bigg (\tan \Bigg (\sqrt{\frac{l _1}{8}} (\zeta +u )\Bigg )-\cot \Bigg (\sqrt{\frac{l _1}{8}} (\zeta +u )\Bigg )\Bigg ), \end{aligned}$$

24 $$\begin{aligned} {\mathcal {J}}_{16}(\zeta )= & {} \frac{\sqrt{-\frac{l _1}{2 l _2}} \Bigg (\sqrt{E_1^2-E_2^2}-{\mathfrak {A}}_1 \cos \Bigg (\sqrt{2 l _1} (\zeta +u )\Bigg )\Bigg )}{E_2+{\mathfrak {A}}_1 \sin \Bigg (\sqrt{2 l _1} (\zeta +u )\Bigg )}, \end{aligned}$$

25 $$\begin{aligned} {\mathcal {J}}_{17}(\zeta )= & {} \frac{\sqrt{-\frac{l _1}{2 l _2}} \cos \Bigg (\sqrt{2 l _1} (\zeta +u )\Bigg )}{\sin \Bigg (\sqrt{2 l _1} (\zeta +u )\Bigg )-1}. \end{aligned}$$

Cluster 6. When $$ l _0=0,~l _1>0,$$ we acquire

26 $$\begin{aligned} {\mathcal {J}}_{18}(\zeta )= & {} \frac{4 l _1 e^{\sqrt{l _1} (\zeta +u )}}{e^{2 \sqrt{l _1} (\zeta +u )}-4 l _1 l _2}, \end{aligned}$$

27 $$\begin{aligned} {\mathcal {J}}_{19}(\zeta )= & {} \frac{4 l _1 e^{\sqrt{l _1} (\zeta +u )}}{1-4 l _1 l _2 e^{2 \sqrt{l _1} (\zeta +u )}}. \end{aligned}$$

Cluster 7. When $l _0=0,~l _1=0~\text {and}~l _2>0$, we acquire

28 $$\begin{aligned} {\mathcal {J}}_{20}(\zeta )= & {} \frac{1}{\sqrt{l _2} (\zeta +u )}, \end{aligned}$$

29 $$\begin{aligned} {\mathcal {J}}_{21}(\zeta )= & {} \frac{i}{\sqrt{l _2} (\zeta +u )}. \end{aligned}$$

Step 3. By substituting Eq. (7) into Eq. (6) and taking second-order derivatives of Eq. (7) using Eq. (8), we obtain a polynomial with a power of ${\mathcal {J}}(\zeta )$.

Step 4. Derive the parameters of ${\mathcal {J}}(\zeta )$ with same power and setting them to zero. This algebraic system of equation will solve for ${\mathcal {H}}_{0},~ {\mathcal {H}}_{n}$     ($n=1,2,3,...$).

Step 5. Finally, we utilize Mathematica software to compute this system of algebraic equations and derive the results of unknown parameters. The solution of Eq. (6) is obtained by plugging in these parameter values. The proposed technique facilitates for accurate solutions to NLEEs.

## Mathematical analysis

In present part, we show the capability and accuracy of our suggested approach. This approach enables us to obtain a soliton solution for three-dimensional generalized KP model. Now, using Eq. (5) into Eq. (3), we can transform it into *NLODEs* such as

30 $$\begin{aligned} \left( g_3^2+g_2 g_4++g_3 g_4+g_1 \left( g_4-3 g_2\right) \right) {\mathcal {Q}}(\zeta )-g_1^2 \left( g_1 g_2 {\mathcal {Q}}''(\zeta )+3 {\mathcal {Q}}(\zeta )\right) =0. \end{aligned}$$

We obtain $M=1$ by applying the balance principle in Eq. (30). Therefore the solution provided in Eq. (7) using $j=1$ becomes as

31 $$\begin{aligned} {\mathcal {Q}}(\zeta )={\mathcal {H}}_1 {\mathcal {J}}(\zeta )+{\mathcal {H}}_0. \end{aligned}$$

Coefficients of similar powers ${\mathcal {Q}}(\zeta ))^{d}$ are equated in which $d=0,1,2,3,...$. After integrating Eq. (31) into Eq. (30) and proceeding this system through a significant calculation, we arrive at the following Family and solutions. This procedure results in a set of algebraic formulas.

Family-1:

32 $$\begin{aligned} \begin{aligned} \left\{ g_3\rightarrow \sqrt{g_2} \sqrt{g_4},~{\mathcal {H}}_0\rightarrow 0,~{\mathcal {H}}_1\rightarrow -\sqrt{3 g_1^2-g_1 g_4},~l_1\rightarrow \frac{2 \Bigg (3 g_1 g_2-g_4 g_2+g_1 g_4\Bigg )}{g_1^3 g_2}\right\} . \end{aligned} \end{aligned}$$

Based on the analysis, Family 1 is satisfied with the following solutions.

33 $$\begin{aligned} {\mathcal {P}}_{1,1}= & {} -\sqrt{2} \sqrt{3 g_1^2-g_1 g_4} \sqrt{-\frac{3 g_1 g_2-g_4 g_2+g_1 g_4}{g_1^3 g_2 l_2}} \text {sech}\Bigg (\sqrt{2} \sqrt{\frac{3 g_1 g_2-g_4 g_2+g_1 g_4}{g_1^3 g_2}}\nonumber \\{} & {} \Bigg (-g_4 t+g_1 x+g_2 y+\sqrt{g_2} \sqrt{g_4} z+u\Bigg )\Bigg ), \end{aligned}$$

34 $$\begin{aligned} {\mathcal {P}}_{1,2}= & {} -\sqrt{2} \sqrt{3 g_1^2-g_1 g_4} \sqrt{\frac{3 g_1 g_2-g_4 g_2+g_1 g_4}{g_1^3 g_2 l_2}} \text {csch}\Bigg (\sqrt{2} \sqrt{\frac{3 g_1 g_2-g_4 g_2+g_1 g_4}{g_1^3 g_2}} \nonumber \\{} & {} \Bigg (-g_4 t+g_1 x+g_2 y+\sqrt{g_2} \sqrt{g_4} z+u\Bigg )\Bigg ), \end{aligned}$$

35 $$\begin{aligned} {\mathcal {P}}_{1,3}= & {} -\frac{4 \sqrt{2} \sqrt{3 g_1^2-g_1 g_4} \sqrt{\frac{3 g_1 g_2-g_4 g_2+g_1 g_4}{g_1^3 g_2}} k_1}{\Bigg (4 k_1^2-l_2\Bigg ) \sinh \Bigg (\sqrt{2} \sqrt{\frac{3 g_1 g_2-g_4 g_2+g_1 g_4}{g_1^3 g_2}} \Bigg (-g_4 t+g_1 x+g_2 y+\sqrt{g_2} \sqrt{g_4} z+u\Bigg )\Bigg )} \nonumber \\{} & {} +\frac{4 \sqrt{2} \sqrt{3 g_1^2-g_1 g_4} \sqrt{\frac{3 g_1 g_2-g_4 g_2+g_1 g_4}{g_1^3 g_2}} k_1}{\Bigg (4 k_1^2-l_2\Bigg ) \cosh \Bigg (\sqrt{2} \sqrt{\frac{3 g_1 g_2-g_4 g_2+g_1 g_4}{g_1^3 g_2}} \Bigg (-g_4 t+g_1 x+g_2 y+\sqrt{g_2} \sqrt{g_4} z+u\Bigg )\Bigg )}, \end{aligned}$$

36 $$\begin{aligned} {\mathcal {P}}_{1,4}= & {} -\sqrt{3 g_1^2-g_1 g_4} \sqrt{-\frac{3 g_1 g_2-g_4 g_2+g_1 g_4}{g_1^3 g_2 l_2}} \tanh \Bigg (\sqrt{-\frac{3 g_1 g_2-g_4 g_2+g_1 g_4}{g_1^3 g_2}} \nonumber \\{} & {} \Bigg (-g_4 t+g_1 x+g_2 y+\sqrt{g_2} \sqrt{g_4} z+u\Bigg )\Bigg ), \end{aligned}$$

37 $$\begin{aligned} {\mathcal {P}}_{1,5}= & {} \sqrt{3 g_1^2-g_1 g_4} \sqrt{-\frac{3 g_1 g_2-g_4 g_2+g_1 g_4}{g_1^3 g_2 l_2}} \Bigg (-\coth \Bigg (\sqrt{-\frac{3 g_1 g_2-g_4 g_2+g_1 g_4}{g_1^3 g_2}} \nonumber \\{} & {} \Bigg (-g_4 t+g_1 x+g_2 y+\sqrt{g_2} \sqrt{g_4} z+u\Bigg )\Bigg )\Bigg ), \end{aligned}$$

38 $$\begin{aligned} {\mathcal {P}}_{1,6}= & {} -\sqrt{3 g_1^2-g_1 g_4} \sqrt{-\frac{3 g_1 g_2-g_4 g_2+g_1 g_4}{g_1^3 g_2 l_2}} \Bigg (\tanh \Bigg (2 \sqrt{-\frac{3 g_1 g_2-g_4 g_2+g_1 g_4}{g_1^3 g_2}} \nonumber \\{} & {} \Bigg (-g_4 t+g_1 x+g_2 y+\sqrt{g_2} \sqrt{g_4} z+u\Bigg )\Bigg )+i \text {sech}\Bigg (2 \sqrt{-\frac{3 g_1 g_2-g_4 g_2+g_1 g_4}{g_1^3 g_2}} \nonumber \\{} & {} \Bigg (-g_4 t+g_1 x+g_2 y+\sqrt{g_2} \sqrt{g_4} z+u\Bigg )\Bigg )\Bigg ), \end{aligned}$$

39 $$\begin{aligned} {\mathcal {P}}_{1,7}= & {} -\frac{1}{2} \sqrt{3 g_1^2-g_1 g_4} \sqrt{-\frac{3 g_1 g_2-g_4 g_2+g_1 g_4}{g_1^3 g_2 l_2}} \Bigg (\tanh \Bigg (\frac{1}{2} \sqrt{-\frac{3 g_1 g_2-g_4 g_2+g_1 g_4}{g_1^3 g_2}} \nonumber \\{} & {} \Bigg (-g_4 t+g_1 x+g_2 y+\sqrt{g_2} \sqrt{g_4} z+u\Bigg )\Bigg )+i \coth \Bigg (\frac{1}{2} \sqrt{-\frac{3 g_1 g_2-g_4 g_2+g_1 g_4}{g_1^3 g_2}}\nonumber \\{} & {} \Bigg (-g_4 t+g_1 x+g_2 y+\sqrt{g_2} \sqrt{g_4} z+u\Bigg )\Bigg )\Bigg ), \end{aligned}$$

40 $$\begin{aligned} {\mathcal {P}}_{1,8}= & {} -\frac{\sqrt{3 g_1^2-g_1 g_4} \sqrt{-\frac{3 g_1 g_2-g_4 g_2+g_1 g_4}{g_1^3 g_2 l_2}} \Bigg (\sqrt{e_1^2+e_2^2}-e_1 \cosh \Bigg (2 \sqrt{-\frac{3 g_1 g_2-g_4 g_2+g_1 g_4}{g_1^3 g_2}} \Bigg (\zeta +u\Bigg )\Bigg )\Bigg )}{e_1 \sinh \Bigg (2 \sqrt{-\frac{3 g_1 g_2-g_4 g_2+g_1 g_4}{g_1^3 g_2}} \Bigg (-g_4 t+g_1 x+g_2 y+\sqrt{g_2} \sqrt{g_4} z+u\Bigg )\Bigg )+e_2}, \end{aligned}$$

41 $$\begin{aligned} {\mathcal {P}}_{1,9}= & {} -\frac{\sqrt{3 g_1^2-g_1 g_4} \sqrt{-\frac{3 g_1 g_2-g_4 g_2+g_1 g_4}{g_1^3 g_2 l_2}} \cosh \Bigg (2 \sqrt{-\frac{3 g_1 g_2-g_4 g_2+g_1 g_4}{g_1^3 g_2}} \Bigg (-g_4 t+g_1 x+g_2 y+\sqrt{g_2} \sqrt{g_4} z+u\Bigg )\Bigg )}{\sinh \Bigg (2 \sqrt{-\frac{3 g_1 g_2-g_4 g_2+g_1 g_4}{g_1^3 g_2}} \Bigg (-g_4 t+g_1 x+g_2 y+\sqrt{g_2} \sqrt{g_4} z+u\Bigg )\Bigg )+i}, \end{aligned}$$

42 $$\begin{aligned} {\mathcal {P}}_{1,10}= & {} -\sqrt{2} \sqrt{3 g_1^2-g_1 g_4} \sqrt{-\frac{3 g_1 g_2-g_4 g_2+g_1 g_4}{g_1^3 g_2 l_2}} \sec \Bigg (\sqrt{2} \sqrt{-\frac{3 g_1 g_2-g_4 g_2+g_1 g_4}{g_1^3 g_2}} \nonumber \\{} & {} \Bigg (-g_4 t+g_1 x+g_2 y+\sqrt{g_2} \sqrt{g_4} z+u\Bigg )\Bigg ), \end{aligned}$$

43 $$\begin{aligned} {\mathcal {P}}_{1,11}= & {} -\sqrt{2} \sqrt{3 g_1^2-g_1 g_4} \sqrt{-\frac{3 g_1 g_2-g_4 g_2+g_1 g_4}{g_1^3 g_2 l_2}} \csc \Bigg (\sqrt{2} \sqrt{-\frac{3 g_1 g_2-g_4 g_2+g_1 g_4}{g_1^3 g_2}}\nonumber \\{} & {} \Bigg (-g_4 t+g_1 x+g_2 y+\sqrt{g_2} \sqrt{g_4} z+u\Bigg )\Bigg ), \end{aligned}$$

44 $$\begin{aligned} {\mathcal {P}}_{1,12}= & {} -\sqrt{3 g_1^2-g_1 g_4} \sqrt{\frac{3 g_1 g_2-g_4 g_2+g_1 g_4}{g_1^3 g_2 l_2}} \tan \Bigg (\sqrt{\frac{3 g_1 g_2-g_4 g_2+g_1 g_4}{g_1^3 g_2}} \nonumber \\{} & {} \Bigg (-g_4 t+g_1 x+g_2 y+\sqrt{g_2} \sqrt{g_4} z+u\Bigg )\Bigg ), \end{aligned}$$

45 $$\begin{aligned} {\mathcal {P}}_{1,13}= & {} \sqrt{3 g_1^2-g_1 g_4} \sqrt{\frac{3 g_1 g_2-g_4 g_2+g_1 g_4}{g_1^3 g_2 l_2}} \Bigg (-\cot \Bigg (\sqrt{\frac{3 g_1 g_2-g_4 g_2+g_1 g_4}{g_1^3 g_2}} \nonumber \\{} & {} \Bigg (-g_4 t+g_1 x+g_2 y+\sqrt{g_2} \sqrt{g_4} z+u\Bigg )\Bigg )\Bigg ), \end{aligned}$$

46 $$\begin{aligned} {\mathcal {P}}_{1,14}= & {} \sqrt{3 g_1^2-g_1 g_4} \sqrt{\frac{3 g_1 g_2-g_4 g_2+g_1 g_4}{g_1^3 g_2 l_2}} \Bigg (\tan \Bigg (2 \sqrt{\frac{3 g_1 g_2-g_4 g_2+g_1 g_4}{g_1^3 g_2}} \nonumber \\{} & {} \Bigg (-g_4 t+g_1 x+g_2 y+\sqrt{g_2} \sqrt{g_4} z+u\Bigg )\Bigg )-\sec \Bigg (2 \sqrt{\frac{3 g_1 g_2-g_4 g_2+g_1 g_4}{g_1^3 g_2}} \nonumber \\{} & {} \Bigg (-g_4 t+g_1 x+g_2 y+\sqrt{g_2} \sqrt{g_4} z+u\Bigg )\Bigg )\Bigg ), \end{aligned}$$

47 $$\begin{aligned} {\mathcal {P}}_{1,15}= & {} -\frac{1}{2} \sqrt{3 g_1^2-g_1 g_4} \sqrt{\frac{3 g_1 g_2-g_4 g_2+g_1 g_4}{g_1^3 g_2 l_2}} \Bigg (\tan \Bigg (\frac{1}{2} \sqrt{\frac{3 g_1 g_2-g_4 g_2+g_1 g_4}{g_1^3 g_2}}\nonumber \\{} & {} \Bigg (-g_4 t+g_1 x+g_2 y+\sqrt{g_2} \sqrt{g_4} z+u\Bigg )\Bigg )-\cot \Bigg (\frac{1}{2} \sqrt{\frac{3 g_1 g_2-g_4 g_2+g_1 g_4}{g_1^3 g_2}}\nonumber \\{} & {} \Bigg (-g_4 t+g_1 x+g_2 y+\sqrt{g_2} \sqrt{g_4} z+u\Bigg )\Bigg )\Bigg ), \end{aligned}$$

48 $$\begin{aligned} {\mathcal {P}}_{1,16}= & {} -\frac{\sqrt{3 g_1^2-g_1 g_4} \sqrt{\frac{3 g_1 g_2-g_4 g_2+g_1 g_4}{g_1^3 g_2 l_2}} \Bigg (\sqrt{e_1^2-e_2^2}-e_1 \cos \Bigg (2 \sqrt{\frac{3 g_1 g_2-g_4 g_2+g_1 g_4}{g_1^3 g_2}} \Bigg (\zeta +u\Bigg )\Bigg )\Bigg )}{e_1 \sin \Bigg (2 \sqrt{\frac{3 g_1 g_2-g_4 g_2+g_1 g_4}{g_1^3 g_2}} \Bigg (-g_4 t+g_1 x+g_2 y+\sqrt{g_2} \sqrt{g_4} z+u\Bigg )\Bigg )+e_2}, \end{aligned}$$

49 $$\begin{aligned} {\mathcal {P}}_{1,17}= & {} \sqrt{3 g_1^2-g_1 g_4} \sqrt{\frac{3 g_1 g_2-g_4 g_2+g_1 g_4}{g_1^3 g_2 l_2}} \Bigg (-\cot \Bigg (2 \sqrt{\frac{3 g_1 g_2-g_4 g_2+g_1 g_4}{g_1^3 g_2}} \nonumber \\{} & {} \Bigg (-g_4 t+g_1 x+g_2 y+\sqrt{g_2} \sqrt{g_4} z+u\Bigg )\Bigg )\Bigg ), \end{aligned}$$

50 $$\begin{aligned} {\mathcal {P}}_{1,18}= & {} -\frac{8 \sqrt{3 g_1^2-g_1 g_4} \Bigg (3 g_1 g_2-g_4 g_2+g_1 g_4\Bigg ) \exp \Bigg (\sqrt{2} \sqrt{\frac{3 g_1 g_2-g_4 g_2+g_1 g_4}{g_1^3 g_2}} \Bigg (\zeta +u\Bigg )\Bigg )}{g_1^3 g_2 \Bigg (\exp \Bigg (2 \sqrt{2} \sqrt{\frac{3 g_1 g_2-g_4 g_2+g_1 g_4}{g_1^3 g_2}} \Bigg (\zeta +u\Bigg )\Bigg )-\frac{8 \Bigg (3 g_1 g_2-g_4 g_2+g_1 g_4\Bigg ) l_2}{g_1^3 g_2}\Bigg )}, \end{aligned}$$

51 $$\begin{aligned} {\mathcal {P}}_{1,19}= & {} -\frac{8 \sqrt{3 g_1^2-g_1 g_4} \Bigg (3 g_1 g_2-g_4 g_2+g_1 g_4\Bigg ) \exp \Bigg (\sqrt{2} \sqrt{\frac{3 g_1 g_2-g_4 g_2+g_1 g_4}{g_1^3 g_2}} \Bigg (\zeta +u\Bigg )\Bigg )}{g_1^3 g_2 \Bigg (1-\frac{8 \Bigg (3 g_1 g_2-g_4 g_2+g_1 g_4\Bigg ) l_2 \exp \Bigg (2 \sqrt{2} \sqrt{\frac{3 g_1 g_2-g_4 g_2+g_1 g_4}{g_1^3 g_2}} \Bigg (\zeta +u\Bigg )\Bigg )}{g_1^3 g_2}\Bigg )}, \end{aligned}$$

52 $$\begin{aligned} {\mathcal {P}}_{1,20}= & {} -\frac{\sqrt{3 g_1^2-g_1 g_4}}{\sqrt{l_2} \Bigg (-g_4 t+g_1 x+g_2 y+\sqrt{g_2} \sqrt{g_4} z+u\Bigg )}, \end{aligned}$$

53 $$\begin{aligned} {\mathcal {P}}_{1,21}= & {} -\frac{i \sqrt{3 g_1^2-g_1 g_4}}{\sqrt{-l_2} \Bigg (-g_4 t+g_1 x+g_2 y+\sqrt{g_2} \sqrt{g_4} z+u\Bigg )}. \end{aligned}$$

**Figure 1:**
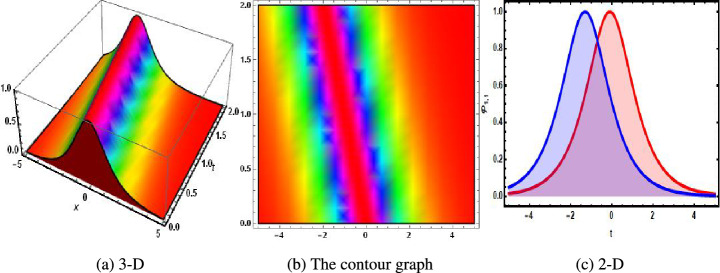
The physical behavior of bright soliton solution of Eq. (33) when $u=1.1.$. (**a**) 3-D, (**b**) the contour graph, (**c**) 2-D.

**Figure 2:**
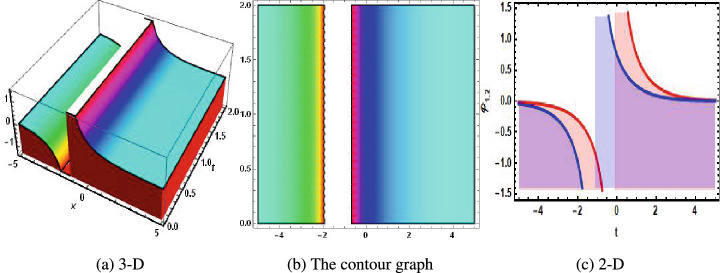
The physical of singular soliton solution of Eq. (34) when $u=1.1.$. (**a**) 3-D, (**b**) the contour graph, (**c**) 2-D.

**Figure 3:**
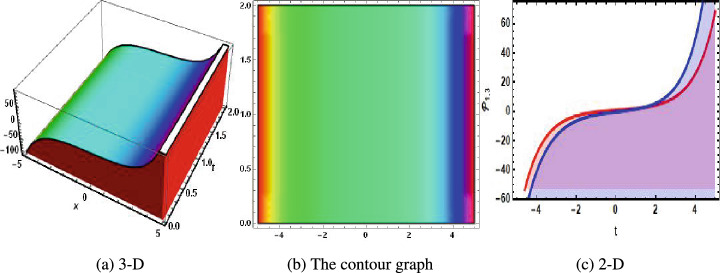
The physical of hyperbolic of solution of Eq. (35) when $u=0.6.$. (**a**) 3-D, (**b**) the contour graph, (**c**) 2-D.

**Figure 4:**
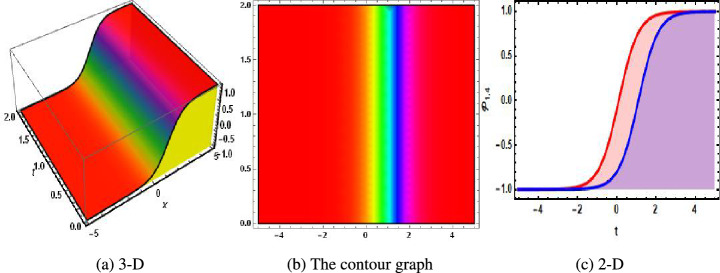
The physical of kink solution of Eq. (36) when $u=0.31.$. (**a**) 3-D, (**b**) the contour graph, (**c**) 2-D.

**Figure 5:**
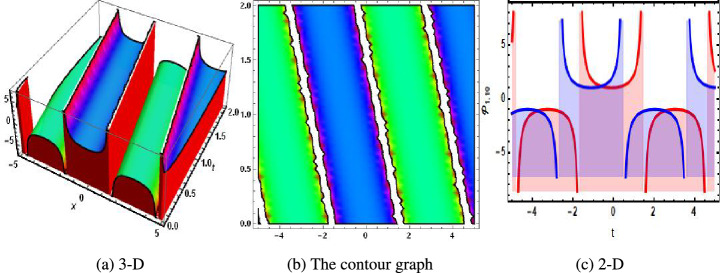
The physical of periodic solution of Eq. (42) when $u=2.1.$. (**a**) 3-D, (**b**) the contour graph, (**c**) 2-D.

**Figure 6:**
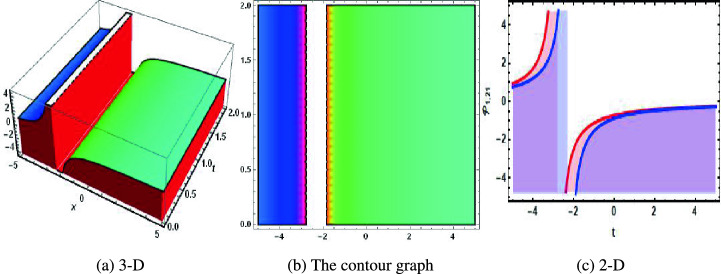
The physical of plane wave solution of Eq. (53) when $u=0.1$. (**a**) 3-D, (**b**) the contour graph, (**c**) 2-D.

## Dynamical system

A dynamical system with complex behavior that changes with time is found to display bifurcation and chaos. A chaotic system behaves in a way that is very sensitive to initial conditions as well as random and unpredictable events. The behavior of the system varies considerably in terms of performance when a parameter is changed during the chaotic^34^. An analysis of the structure of various fundamental systems with dynamics in terms of bifurcation and chaos is presented. When one or more parameters suddenly change, usually as a result of small adjustments, bifurcation takes place.

### Chaotic structure with perturbation

**Figure 7:**
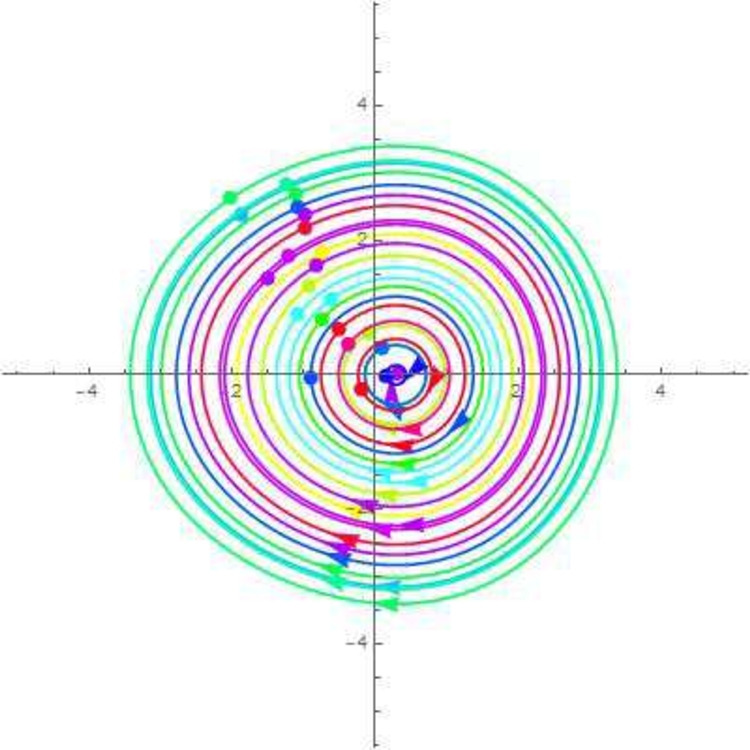
Chaotic behavior of center points of Eq. (54), when $\phi =~0.1$ and $\xi =1$.

**Figure 8:**
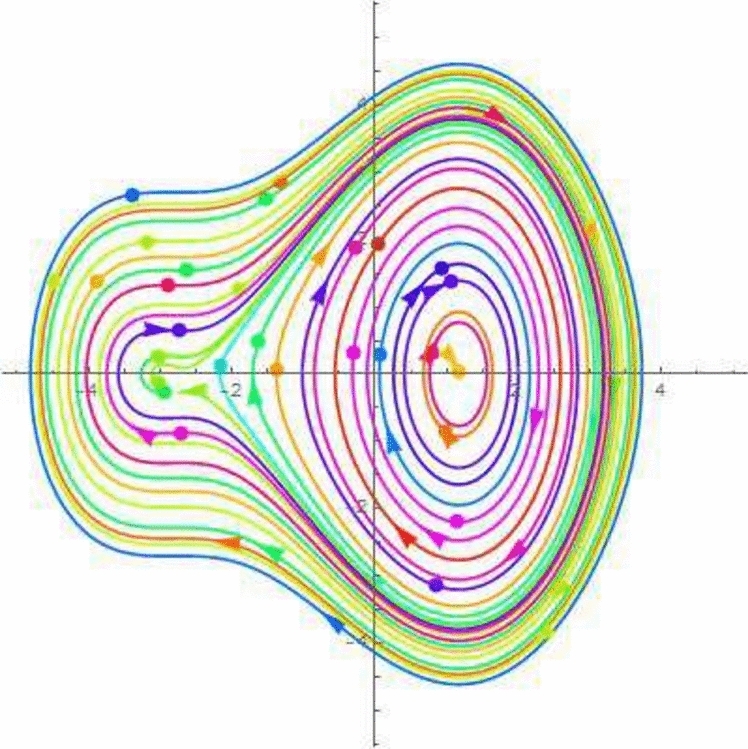
Chaotic behavior of cuspidal points of Eq. (54), when $\phi =~0.4$ and $\xi =3.1$.

**Figure 9:**
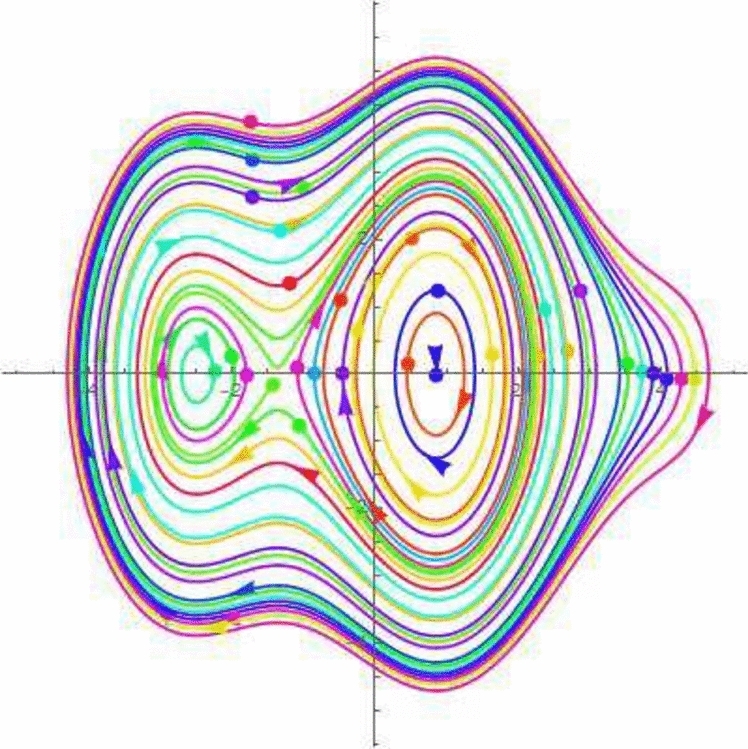
Chaotic behavior of saddle points of Eq. (54), when $\phi =~0.7$ and $\xi =-0.1$.

**Figure 10:**
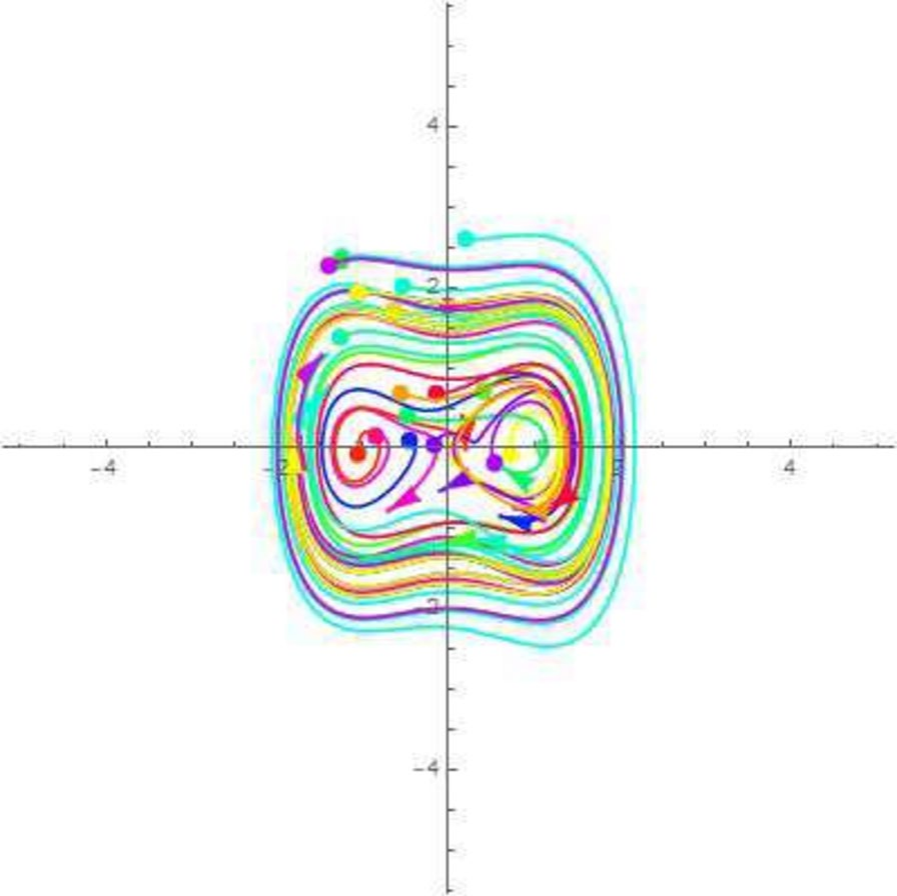
Chaotic behavior of saddle and center points of Eq. (54), when $\phi =~1.1$ and $\xi =0.2$.

A disturbed systems with dynamics experiences external perturbations or changes in its features. Bifurcation and chaos are two results of plungations that have the potential to significantly modify the behaviour of the structure. Chaos in a dynamical framework is characterized by bounded parameters that exhibit differences but do not follow a periodic or quasi-periodic pattern. This process is a significant factor in carrying such systems towards chaotic actions. The perturbation analysis is a theoretical framework for analyzing minor disturbances in a dynamic structure. Using Galilean transformation and perturbation component $\phi \cos (\xi F)$ turn the *NLODEs* from Eq. (30) into a dynamical system. For the perturbation-term dynamical system, it becomes

54 $$\begin{aligned} {\mathcal {Q}}'&={\mathfrak {A}}_1, \nonumber \\ {\mathcal {Q}}''&=\frac{1}{g_1^3g_2}\Bigg ((g_3^2+g_2g_4+g_1(g_4-3g_2)){\mathcal {Q}}(\zeta )-3g_1^2(g_1g_2){\mathcal {Q}}(\xi )\Bigg )+\phi \cos (\zeta F)={\mathfrak {A}}_2, \end{aligned}$$

in which $\xi$ and $\phi$ show amplitude and frequency component. By selecting suitable parameter values, the 2D chaotic structure of Eq. (54) is illustrated in Figs. 7, 8, 9 and 10.

### Numerical simulation

By precisely characterising the system, we use phase pictures and numerical simulations to thoroughly analyse the dynamical behaviour of the system. This involves recording the initial conditions, any relevant parameters, and the governing differential equations. To solve the differential equations, we perform some numerical simulations. We can utilize a variety of numerical approaches, such as Runge-Kutta methods, Euler’s method, or more advanced methods, depending on the system. This can estimate the greatest Lyapunov exponent using numerical methods. Chaos is indicated by a high Lyapunov exponent, whereas regular (periodic or quasi-periodic) behaviour is suggested by a negative exponent.

## Discussions and results

In 2016, Wazawaz^29^ utilized the Hirota’s direct scheme to obtain some novel soliton solutions of multiple cases of this model. In present work, we apply MSSEA approach to identify the dark, solitary, periodic, and rational solutions of this generalized (3 + 1)-diemnsional KP model. The 2D chaotic structures of governing system with perturbation terms are also obtained. The following points illustrate the physical illustration of acquired results.

- The component ${\mathcal {P}}_{1,1}$ of Eq. (33) illustrates the bright soliton solution.
- The component ${\mathcal {P}}_{1,2}$ of Eq. (34) illustrates the singular soliton solution.
- The component ${\mathcal {P}}_{1,3}$ of Eq. (35) illustrates the hyperbolic soliton solution.
- The component ${\mathcal {P}}_{1,4}$ of Eq. (36) illustrates the dark soliton solution.
- The component ${\mathcal {P}}_{1,5}$ of Eq. (37) illustrates the singular soliton solution.
- The components ${\mathcal {P}}_{1,6}$ and ${\mathcal {P}}_{1,7}$ of Eqs. (38) and (39) illustrate the combo of dark and bright soliton solution.
- The components ${\mathcal {P}}_{1,8}$ and ${\mathcal {P}}_{1,9}$ of Eqs. (40) and (41) illustrate the hyperbolic soliton solution.
- The components ${\mathcal {P}}_{1,10},~{\mathcal {P}}_{1,11},~{\mathcal {P}}_{1,12},~{\mathcal {P}}_{1,13},~{\mathcal {P}}_{1,14},~{\mathcal {P}}_{1,15},~{\mathcal {P}}_{1,16}~\text {and}~{\mathcal {P}}_{1,17}$ of Eqs. (42–49) illustrate the periodic solution.
- The components ${\mathcal {P}}_{1,18}$ and ${\mathcal {P}}_{1,19}$ of Eqs. (50 and 51) illustrate the exponential solution.
- The components ${\mathcal {P}}_{1,20}$ and ${\mathcal {P}}_{1,21}$ of Eqs. (52 and 53) illustrate the rational solution. Figures 1, 2, 3, 4, 5 and 6 show the structure of soliton solutions at $g_1=0.3,~g_2=0.8,~g_3=0.6,~g_4=1.1,~u=0.4,~l_2=0.1,~y=0.1,~z=0.2$, whereas Figs. 7, 8, 9 and 10 depicts the structure of every potential description of a chaotic structure to a nonlinear dynamical system.
- The Fig. 1 illustrate the bright solutions. Bright soliton refers to the solitary waves.
- The Fig. 2 illustrate the singular soliton solutions. Peakons are the cases for a single solitary wave solution in which peaks contain a discontinuous first derivative.
- The Fig. 3 illustrate the hyperbolic solutions. The hyperbolic has limited (compact) stability. The Fig. 4 illustrate the kink solutions. The Fig. 5 illustrate the periodic solutions. The Fig. 6 illustrate the plane wave solutions. The Figs. 7, 8, 8, 9 and 10 illustrate the graphical behavior of chaotic structures.

Sharp transitions and discontinuities are characteristics of singular solutions, which have a wide range of applications in various domains. They simulate turbulence and wave breaking in fluid dynamics. They explain how optical shocks occur in nonlinear media in optics. They are used in plasma physics to investigate fast variations in electric fields or plasma densities, and they are also useful in hydrodynamics to comprehend gravitation and other fast pressure changes. Furthermore, these solutions provide important insights into complicated nonlinear phenomena and are essential in geophysical flows, nonlinear wave propagation in lattices, and even in general relativity for analyzing space-time singularities and gravitational wave dynamics.

## Conclusion

In this work, we have examined the performance of MSSEA to extract the soliton solutions and qualitative analysis for a generalized KP model in the (3 + 1)-dimensional form. The chaotic structures with perturbation terms help us to understand the planar dynamical system. We provide some illustrations of these results that includes dark, solitary, periodic, and rational solutions. We believe that this new exciting insights may provide a better light on the movement of liquid ripples in mathematical modeling. Besides, we also explained the mechanism of fluid that relies on physical elements in action. The derived results show that the suggested approach is a dynamic and effective quantitative mechanism for a broad area of nonlinear wave challenges in mathematical science and technology, and and numerous other nonlinear disciplines. The modified Sardar sub-equation approach is a highly effective tool for addressing nonlinear PDEs, offering precise solutions that can provide valuable understanding of the fundamental physical phenomena. Nevertheless, its applicability is restricted by the specific forms of equations, particularly for higher-dimensional PDEs, which may be challenging and time-consuming to compute. This approach under consideration yields a large number of solitons and has the potential to be effectively utilized for nonlinear problems that model a range of natural phenomena. In the future, we intend to explore some more techniques that could potentially uncover novel optical soliton solutions for the current model involving different fractional derivatives. This will lay the groundwork for a compelling comparison of our findings with those generated from this approach, expanding the scope of study in this exciting field.

## Data Availability

All data generated or analysed during this study are included in this published article.
